# Feasibility and Oncological Outcome of Preoperative Chemoradiation With IMRT Dose Intensification for Locally Advanced Esophageal and Gastroesophageal Cancer

**DOI:** 10.3389/fonc.2021.626275

**Published:** 2021-02-18

**Authors:** Roberto Innocente, Federico Navarria, Roberto Petri, Elisa Palazzari, Massimo Vecchiato, Jerry Polesel, Antonio Ziccarelli, Antonio Martino, Paolo Ubiali, Dino Tonin, Andrea Lauretta, Claudio Belluco, Luisa Foltran, Angela Buonadonna, Arben Lleshi, Carlotta Benedetta Colombo, Loredana Barresi, Marco Gigante, Giovanni Franchin, Antonino De Paoli

**Affiliations:** ^1^ Radiation Oncology Department, Centro di Riferimento Oncologico di Aviano (CRO) IRCCS, Aviano, Italy; ^2^ General Surgery Department, Azienda Sanitaria Universitaria Friuli Centrale (ASU FC), Udine, Italy; ^3^ Unit of Cancer Epidemiology, Centro di Riferimento Oncologico di Aviano (CRO) IRCCS, Aviano, Italy; ^4^ General Surgery Department Azienda per l’Assistenza Sanitaria n. 5 Friuli Occidentale, Pordenone, Italy; ^5^ Oncological Surgery Department, Centro di Riferimento Oncologico di Aviano (CRO) IRCCS, Aviano, Italy; ^6^ Medical Oncology Department Centro di Riferimento Oncologico di Aviano (CRO) IRCCS, Aviano, Italy; ^7^ Nuclear Medicine Department, Centro di Riferimento Oncologico di Aviano (CRO) IRCCS, Aviano, Italy; ^8^ Medical Physics Department, Centro di Riferimento Oncologico di Aviano (CRO) IRCCS, Aviano, Italy

**Keywords:** esophageal cancer, gastroesophageal junction cancer, intensity-modulated radiotherapy, simultaneous integrated boost, dose intensification

## Abstract

**Purpose:**

To explore the feasibility and efficacy of a dose intensification with Intensity Modulated Radiation Therapy and Simultaneous Integrated Boost (IMRT-SIB) in locally advanced esophageal and gastroesophageal cancer (GEJ).

**Methods and Materials:**

We retrospectively analyzed a series of 69 patients with esophageal or GEJ cancer treated at our Institute, between 2016 and 2019, with preoperative IMRT and SIB up to 52.5–54 Gy in 25 fractions in 5 weeks and concurrent carboplatin (AUC2) and paclitaxel (50 mg/m^2^), as in the CROSS regimen.

**Results:**

All patients completed the planned IMRT–SIB program with a median of four (range 1–5) cycles of concurrent paclitaxel/carboplatin. Compliance to IMRT–SIB was 93%, whereas 54% of patients received four to five cycles and 87% at least three cycles of concurrent carboplatin/paclitaxel. Grade 3 toxicity was reported in 19% of patients. Complete clinical response (cCR) was achieved in 48%, and 13% had disease progression after chemoradiation (CRT). Overall, 49% of patients underwent surgery; reasons for non-operation included cCR in cervical tumor location (10%) or cCR and patient decision (13%). A pathologic complete response (pCR) was achieved in 44% of resected patients. Postoperative complications and mortality rates were 21 and 6%, respectively. At a median follow-up of 12 months (6–25), 2-year overall and progression-free (PFS) survival rates were 81 and 54%, respectively. No difference in PFS by histologic type in operated patients was reported. Non-operated cCR patients had higher PFS, including cervical locations and selected cCR patients who decided for non-operation (75 *vs* 30%, p < 0.01).

**Conclusion:**

The study reported favorable results in safety and feasibility of the IMRT–SIB dose intensification in our preoperative CRT program. The toxicity was acceptable, allowing a high compliance to intensified radiation doses with dose reduction of concurrent paclitaxel/carboplatin in some patients. The high rate of cCR and pCR suggested this intensified program is effective in the preoperative CRT and, for selected responsive patients, in the non-operative approach to esophageal and GEJ cancer. The 2-year survival rates were promising. A prospective study is being planned to confirm these observations.

## Introduction

Ranked the eighth most common cancer in incidence and the sixth leading cause of cancer-related death worldwide, esophageal cancer remains a major global health problem (1, 2). Epidemiological changes have occurred in the last decades with an increasing incidence of adenocarcinoma (AC) in distal esophagus and gastro-esophageal junction (GEJ) in Western Countries, whereas squamous cell carcinoma (SCC) remains the most common histology in Eastern Europe and Asia. Risk factors associated with AC include high rates of gastroesophageal reflux disease, obesity, and Barrett esophagus (3).

Combined modality treatment including preoperative chemoradiation (CRT) followed by radical surgery has become the standard of care for most patients with localized clinical stage T2–T3, N0-1 resectable disease. The more recent published results of the CROSS trial comparing preoperative weekly paclitaxel/carboplatin concurrent with radiation therapy of 41.4 Gy *versus* surgery alone (4) reported a survival benefit, thus confirming the previous indications of smaller phase III trials (5–7) and meta-analysis (8). Tolerance to preoperative CRT in the CROSS trial was well acceptable, and most patients completed the planned treatment. Importantly, preoperative CRT did not significantly increase the postoperative morbidity or mortality rate nor did it negatively impact the postoperative health-related quality of life compared to surgery alone.

The CROSS regimen increasingly became a reference preoperative treatment for locally advanced esophageal and GEJ cancer in the clinical practice; this regimen also promoted an investigational interest in refining the treatment schedule, in particular radiation dose and modality, to further improve disease control and survival. Modified-CROSS regimens with a radiation dose higher than 41.4 Gy have been investigated with conflicting results (9, 10). At our Institute we explored a modified-CROSS regimen including a moderate radiation dose intensification with IMRT and simultaneous integrated boost (SIB) in a cohort of patients with esophageal and GEJ cancer. We report the analysis on feasibility and oncological outcomes of this new treatment approach.

## Material and Methods

### Patient Selection

A series of 69 patients with potentially resectable, cT2–T4 or N1–2, M0, histologically confirmed SCC or AC of the esophagus or GEJ (Siewert I–II) were selected for this preoperative CRT program with intensified an IMRT–SIB approach at our Institution and retrospectively reviewed. This study was included in a clinical research program on gastric and gastroesophageal cancer at our Institute and approved by the Institutional Review Board (CRO-2008-26). All clinical cases were discussed by the institutional multidisciplinary team (MDT) and a signed written informed consent was obtained from each patient.

Baseline evaluation included clinical history and physical examination, hematologic and biochemical tests; pulmonary-function tests, upper gastrointestinal endoscopy with biopsy and endoscopic ultrasonography (EUS); computed tomography of the neck, chest and upper abdomen. A bronchoscopy examination was performed for middle esophagus locations and positron emission tomography/computed tomography (PET-CT) was also included in the staging procedures.

### Treatment

All patients received preoperative concurrent CRT which was followed by MDT re-evaluation for surgery. A non-operative approach was also considered for surgically critical tumor locations (*i.e.* cervical esophagus) and in carefully selected complete responding patients who decided for non-operation. As in the CROSS regimen, chemotherapy (CT) consisted of a weekly administration of paclitaxel 50 mg/m^2^ and carboplatin AUC2 given intravenously with a total infusion time of 2 h for 5 weeks on days 1, 8, 15, 22, and 29. whereas an intensified A radiation dose of radiation of 45 Gy/25 fractions/5 weeks (1,8 Gy/fraction) was provided with sliding-window IMRT or VMAT technique to the gross primary tumor, involved nodes, elective regional nodes at risk, and a simultaneous integrated boost SIB up to 52.5–54 Gy to the gross tumor volume (GTV) and involved nodes only. Patients received computed tomography simulation and treatment in supine position with knee support for the legs and with the arms lifted above the head, using an arm-immobilization system in mid-thoracic and lower localizations, and a thermoplastic mask with shoulder immobilization for cervical and upper thoracic localizations. Since respiratory motion may be significant in lower localizations, 4D-CT planning was used to define the internal target volume (ITV) according to the observed motion. Patients were treated in free-breathing and were instructed to avoid food intake 2 to 3 h before simulation and treatment.

**Figure 1:**
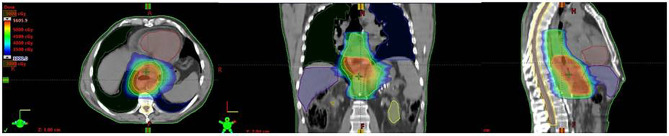
An example of the dosimetric plan with IMRT-SIB of 45–52.5 Gy for EGJ (Siewert 1) adenocarcinoma and normal tissue dose limits. This example demonstrates the coverage of PTV 52.5 Gy (orange) including GTV and PTV 45 Gy (blue), and respect of OARs (Right Lung: green, Left Lung: blue; Hearth: brown, Liver: purple, Spinal cord: light orange and Spinal cord prv: yellow).

The GTV was contoured using PET–CT fusion scans. PET positive lymph nodes where included in the GTV. SIB was limited to GTV and dose was up to 50–52.5 Gy/25fractions/5weeks (2–2.10 Gy/fraction) for thoracic and GEJ locations, and up to 54 Gy/25 fractions (2.16 Gy/fraction) for cervical esophageal cancer locations. The clinical target volume (CTV) was defined by expanding the GTV by 3–5 cm superiorly and inferiorly and 1 cm radially. The CTV was then manually refined on the basis of the patient’s anatomy and tumor location. In particular, for inferior esophagus and GEJ tumors, optimized target volumes were delineated for each Siewert’s type involvement including the supradiaphragmatic, and proximal gastric with celiac lymph node stations (11), resulting in a significant variation in target volumes contoured, volume extension and organs at risk (OARs) involved. The planning target volume (PTV) was created by a uniform expansion of 0.5 cm around the CTV, including the ITV for EGJ tumors as defined above. OARs for treatment planning included lungs, heart, uninvolved esophagus and stomach, liver, kidneys, and spinal cord. Radiation dose was prescribed to the PTVs according to the International Commission on Radiation Units & Measurements (ICRU) criteria (12) and normal tissue dose constraints were defined according to European Organisation for Research and Treatment of Cancer- Radiation Oncology Group (EORTC-ROG) (11) and NCCN (13) guidelines with priority to maximum spinal cord dose and volumetric heart and lung dose (14). An example of the dosimetric plan for EGJ adenocarcinoma (Siewert 1) and normal tissue dose limits is reported in **Figure 1**; further details in OAR dose constraints and DVH are reported in **Table S1** and **Figure S1** (Supplementary documents) Image-guided radiation therapy (IGRT) were used for treatment delivery in all patients. Clinical and nutritional monitoring of patients with hematologic–biochemical test were planned weekly and CRT dose modifications were provided, when needed.

After CRT, a complete re-staging including upper gastrointestinal endoscopy with biopsy, EUS, computed tomography of the neck, chest, upper abdomen, and PET–CT was planned at 6–7 weeks to assess treatment response and M0 status. After re-staging, the patients were evaluated for surgery by the MDT. Surgery was usually planned 8 to 10 weeks after CRT. Most patients had been referred to our Institute from the Surgical Department dedicated to esophageal disease of the University Hospital of Udine, where they were operated on, usually with a minimally invasive approach. Other patients underwent surgery at our Institute or at outside hospitals with the more traditional open approach.

### Data Collection

Medical records of all patients with SCC or AC of the esophagus or GEJ treated with the modified-CROSS regimen from February 2016 to October 2019 were retrospectively reviewed. Patient characteristics including age, gender, ECOG performance status, pre- and post-CRT weight and comorbidities were recorded. Initial tumor characteristics including histology, tumor location with Siewert classification for EGJ AC, pre- and post-CRT clinical stage based on computed tomography, EUS, PET–CT were reviewed. Clinical response evaluation was made according to Response evaluation criteria in solid tumors (RECIST) criteria (15) and complete clinical response (cCR) was defined as complete disappearance of tumor and nodal involvement at computed tomography, EUS when technically feasible, and at gastroscopy with negative biopsy. Parameters analyzed in PET–CT response evaluation included pre- and post-therapy SUVmax, according to Singh et al. (16). Treatment characteristics, including regimen and number of neoadjuvant CT cycles, if administered, number of concurrent CT and IMRT–SIB dose, toxicities, dose attenuation or treatment interruption, as well as surgery performed, pathological data including pathological response and Tumor Regression Grade (TRG) according to Mandard et al. (17), postoperative morbidity and mortality (<30 days) were recorded. Clinical multidisciplinary follow-up data, with site and date of recurrences were also registered.

### Statistical Analysis

Socio-demographic and clinical characteristics were described using median values (with interquartile range) or percentages. For each patient, the time at risk was calculated from the end of CRT to the recurrence, death or end of follow-up, whichever occurred first. The event of interest was death for overall survival and death or recurrence of progression-free survival. The survival probabilities were calculated according to the Kaplan–Meier method and difference between strata were tested through the log-rank test.

## Results

### Patient and Tumor Characteristics

Overall, a cohort of 69 patients with histologically confirmed esophageal or GEJ cancer, treated from February 2016 to October 2019, with the modified-CROSS regimen including an IMRT–SIB dose intensified program were considered in this analysis. Patient and tumor characteristics are reported in **Table 1**. The majority of patients were males (83%) with a median age of 69 years. The GEJ was the most frequent subsite location (35%), equally represented by Siewert type I (tumor epicenter located between 1 and 5 cm above the GEJ) and type II (epicenter 1 cm above and 2 cm below the GEJ). The middle third esophagus was the second most common sub-site (30%) followed by the proximal third (26%) including cervical esophagus (8%). However, AC histology accounted for 45% of cases; 10% of these cases were in the low or middle esophagus. EUS was performed in 64% of patients and PET–CT in 90% of cases. Most patients had stage T3 (80%) and N1 (68%) disease.

**Table 1:** Patient and tumor characteristics.

Characteristics	N. of Patients n = 69	%
**Age (yrs)**		
Median (range)	69 (38–85)	
**Gender**		
Male	57	83
Female	12	17
**Performance Status ECOG**		
0	18	26
1	51	74
**Tumor type**		
Squamous cell carcinoma	40	58
Adenocarcinoma	29	42
**Tumor location**		
Proximal third	19	27
(cervical)*	(7)*	10
Middle third	20	29
Distal third	6	9
Gastroesophageal junction	24	25
Siewert 1	14	
Siewert 2	10	
Siewert 3	–	
**Clinical tumor stage**		
T1	–	–
T2	5	7
T3	55	80
T4	9	13
**Clinical nodal stage**		
N0	9	13
N1	47	68
N2	11	16
NX	2	3

*In parentheses: subsites/overall population.

### Preoperative Chemoradiation, Toxicity, and Clinical Tumor Response

All patients completed the planned intensified IMRT and SIB treatment with 45 Gy to PTV1 (GTV with involved nodes and elective nodal stations) and a median dose of 52.5 Gy (range 50–54Gy) to PTV2 (expanded GTV) and concurrent carboplatin and paclitaxel with a median of four cycles (range 1–5). Five patients had radiation therapy interruption, and a delay or interruption of CT was required in nine (13%) patients after two cycles and in 23 (33%) after three cycles respectively because of grade 3 or persistent grade 2 toxicity (NCI-CTAE criteria, version 4.0) (18). Overall, the compliance to IMRT–SIB dose intensification was 93%, while the compliance to concurrent CT with four or five cycles was 54%; 87% of patients received at least three cycles of CT. Grade 2 toxicity, mainly hematological, occurred in 36 patients (52%). Grade 3 toxicity was hematological in nine patients (13%) and gastrointestinal in three (4%) with severe dysphagia and weight loss >10% requiring enteral/parenteral support. No Grade 4 toxicity was reported.

A selected subset of 15 patients (22%) with more advanced disease, stage T3–4 N1–2, received induction taxane-based (11 patients) or cisplatin-based CT (four patients) with a median of three cycles (range 2–5). Induction CT in this subset of patients was well tolerated with limited Grade 3 toxicity (21%), and the tolerance to subsequent CRT was similar to patients not receiving induction CT. Overall toxicity data and treatment compliance are reported in **Table 2**.

**Table 2:** Preoperative chemoradiation, toxicity, and treatment compliance.

	No of Patients n = 69	%
**Carboplatin/Paclitaxel**		
4–5 cycles	37	54
3 cycles	23	33
1–2 cycles	9	13
Median number of cycles: 4		
**IMRT-SIB dose**		
50 Gy	6	9
52.5 Gy	56	81
54 Gy	7	10
Interruption/Delayed IMRT	5	7
**Induction Chemotherapy** 2–4 cycles (median three cycles)	15	22
**Acute toxicity**		
Grade 3 gastrointestinal	4	6
Grade 3 hematologic	9	13
**Treatment Compliance**		
To radiotherapy	64	93
To concurrent chemotherapy (4–5 cycles)	37	54

Clinical response rate (cCR + cPR) after treatment was achieved in 51 out of 69 patients (74%); 33 (48%) had a cCR with negative biopsy and negative PET–CT. A cCR was achieved in all patients with cervical esophageal cancer who received the IMRT–SIB dose of 54 Gy. Nine patients (13%) had disease progression assessed at restaging; sites of disease progression were local in one patient and distant metastasis in eight patients, respectively. Disease progression was reported in two out of 15 patients (13%) who received also induction CT and in 10 out 54 (19%) who received CRT alone. Details of preoperative CRT response are reported in **Table 3**.

**Table 3:** Clinical response to preoperative chemoradiation.

Clinical response	N. of Patients n = 69	%
Complete response (cCR)	33	48
Partial response (cPR)	18	26
Stable disease (SD)	9	13
Progression disease (PD)	9 (1 local; 8 mets)	13

### Surgery, Pathological Assessment, and Postoperative Complications

Overall, 34 out of 69 (49%) patients underwent surgery. The reasons for non-operation were disease progression in 10 patients (14%), poor general conditions in five (7%), no response to CRT in four (6%), cervical tumor location in seven (10%); nine patients (13%) in cCR decided for non-operation and were followed with active surveillance. The median time between the end of preoperative CRT and surgery was 10 weeks (68 days). Surgical procedures consisted in minimally invasive esophagectomy *via* thoracoscopic approach with patient in prone position (19). Surgery was performed at the regional reference surgical department for esophageal disease in 21 patients, whereas 13 patients underwent a more traditional open esophagectomy at our Institution or other hospitals. Two patients (6%) were evaluated as unresectable at surgery. A median of 20 lymph nodes (4–41) were detected after lymphadenectomy.

An R0 resection was achieved in 30 of 32 resected patients (94%); two patients had R1 resection. A pathologic complete response (pCR-ypT0N0) was reported in 14 patients (44%). TRG1 was reported in 15 patients (47%) including one patient with pT0pN1 stage; in addition, six patients (18%) had microscopic residual disease (TRG2). The pCR was achieved in nine of 14 patients (64%) with SCC and in five out of 18 (28%) with AC histology, respectively. Tumor downstaging was reported in 72% of patients and nodal downstaging in 69%, respectively. Surgery, pathological findings, and reasons for non-operation are summarized in **Table 4**.

**Table 4:** Surgery, pathological findings, and non-operation reasons.

Variables Operated patients	N. of Patients 34	% 49 (SCC 30%; AC 70%)
**Surgical procedure**		
Open Ivor–Lewis esophagectomy	13	35
Minimally invasive esophagectomy	21	59
Explorative only	2	6
**Pathologic Response**	**32**	
Complete Response, pT0N0 (pCR)	14	44 *(SCC 64%; AC 28%)
TRG1	°15	47
TRG2	6	18
TRG3	8	25
TRG4-5	3	9
**R0 Resection**	**30 (32)**	94
** Non-operated patients**	**35**	**51 (SCC 74%; AC 26%)**
**Reasons for non-operation**		
Disease progression	10	14
Non-responders to CRT	4	6
Poor general conditions	5	7
Tumor site (cervical)	7	10
Patient decision	9	13

*In parentheses pCR/operated patient subset histotype; °1 TRG1 patient with pT0N1.

SCC, squamous cell carcinoma; AC, adenocarcinoma; CRT, chemoradiation.

The median intensive-care unit stay was 2 days, and the median postoperative hospital stay was 12 days (range 10–58). Postoperative complications were reported in seven of 33 (21%) operated patients. The 30-day postoperative mortality rate was 6%. Two patients died during hospital stay because of ARDS (one patient) and acute pulmonary embolism (one patient). Another patient died postoperatively on day 58 because of sepsis. Details of postoperative complications are reported in **Table 5**.

**Table 5:** Postoperative complications.

	N. of Patients	%
**Operated patients**	**34**	49
**Complications**	**7**	**21**
Anastomotic leakage	3	9
Pneumonia	2	6
Chylothorax	1	3
Abscess	1	3
≤30-days postoperative mortality	2	6
>30-days postoperative mortality	1	3

### Survival

**Figure 2:**
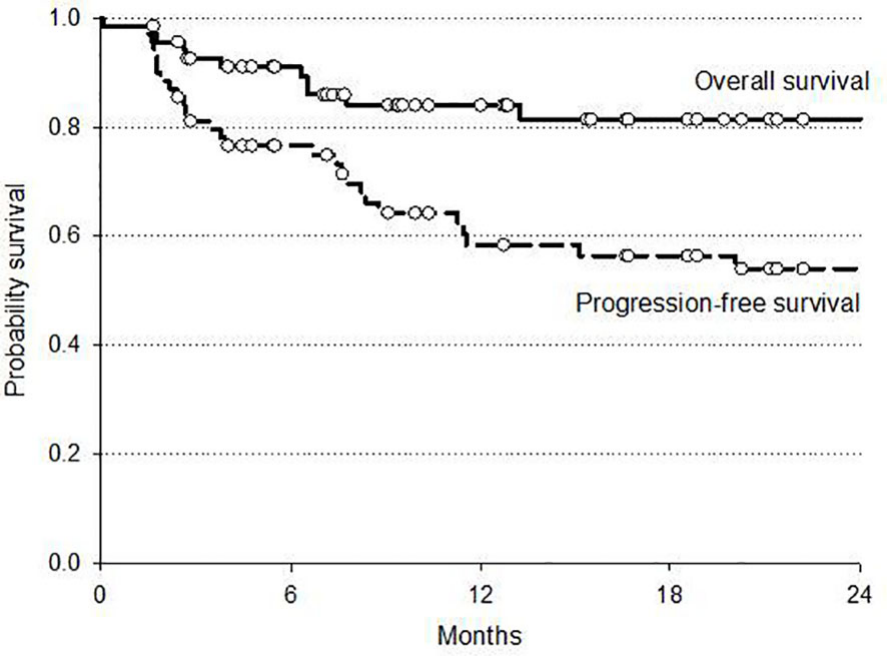
Overall survival and progression-free survival in 69 patients with esophageal-GEJ cancer.

**Figure 3:**
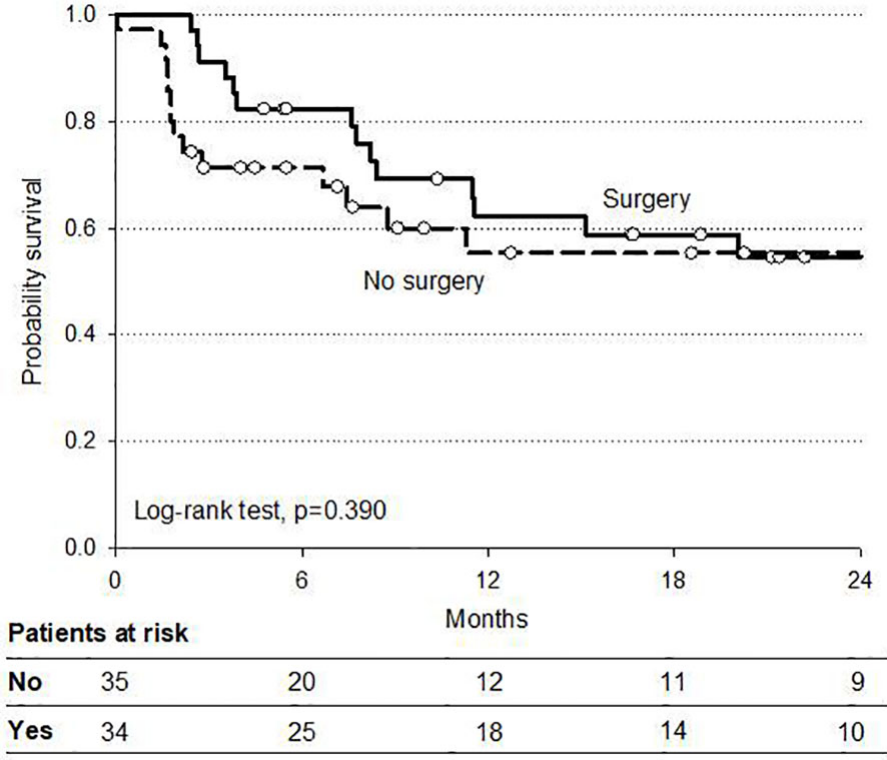
Overall progression-free survival by surgery.

At a median follow-up of 12 months (range 6–25) the 2 year overall survival (OS) and disease-free (DFS) rates were 81 and 54%, respectively. At intention to treat analysis, the 34 patients who received surgery after CRT demonstrated a favorable trend in both OS and DFS compared to non-operated patients. Survival curves are reported in **Figures 2** and **3**.

**Figure 4:**
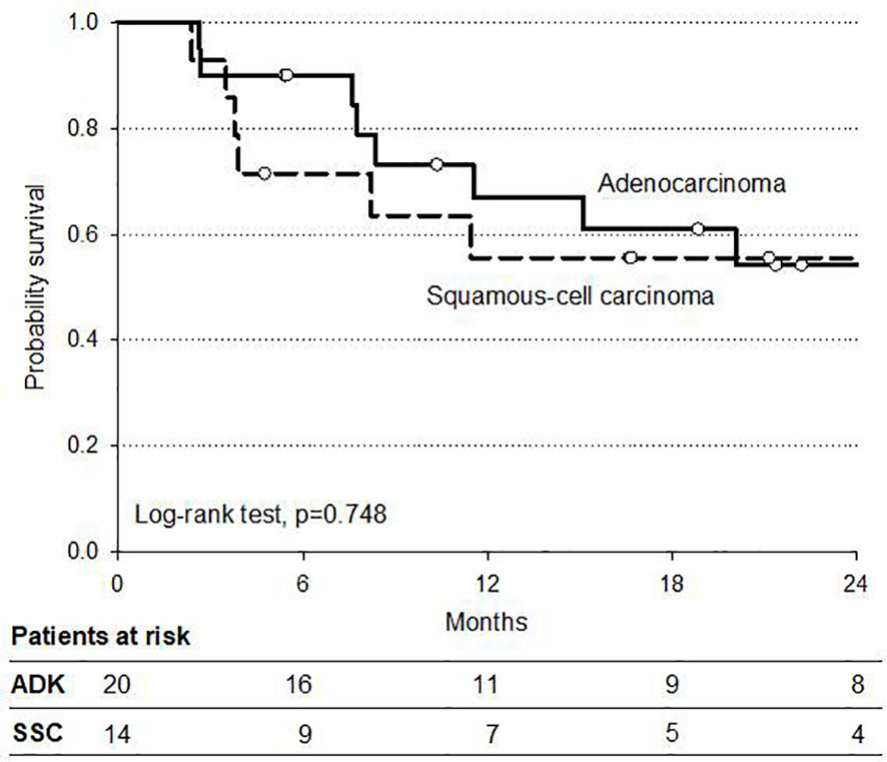
Progression-free survival in 34 operated patients (intention to treat) by histologic type.

Analysis of survival according to histologic subtypes (AC and SCC) in operated patients showed no significant difference in OS (84 and 79%, respectively), although a trend in favor of SCC was reported in DFS (**Figure 4**). Pattern of tumor recurrences after surgery in this setting of operated patients demonstrated a local recurrence rate, as a component of failure of six and 39% of patients had metastatic disease progression alone. Causes of death were disease related in 21 patients (10%) whereas two patients died of other causes. Three patients were lost to follow-up.

**Figure 5:**
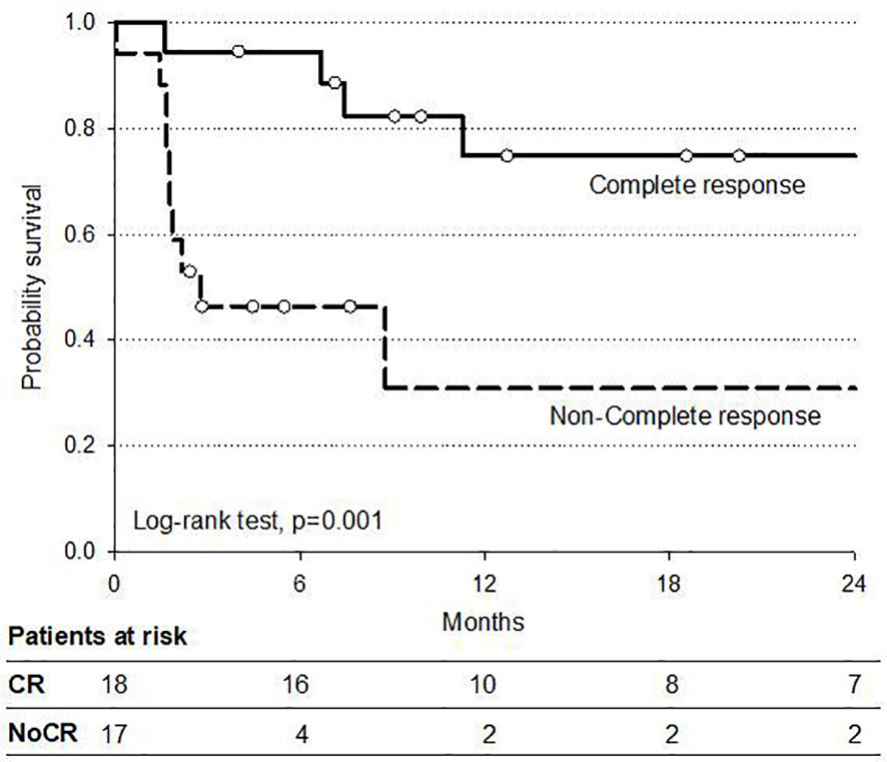
Progression-free survival in 35 non-operated patients by clinical response.

An explorative analysis of survival has been performed also for the 35 non-operated patients to evaluate the outcome of potential different patient subsets. The 2-year DFS of the group of cCR patients, including those with cervical esophageal cancer and the selected cCR patients who decided for non-operation, was 75% compared to 25% of the non-cCR non-operated patients (p < 0.01) (**Figure 5**).

## Discussion

Our study investigated a new preoperative CRT program with a moderately intensified radiotherapy regimen in locally advanced esophageal and GEJ cancer patients. After the publication of the results of the CROSS trial, confirming the benefit of preoperative CRT over surgery alone, the CROSS regimen became a reference neoadjuvant CRT program for locally advanced esophageal and GEJ cancer in the clinical practice (13, 20). In our study, we intensified the radiation dose of the original CROSS regimen because of the reported persistent component of local failure reported in an updated long-term results (21) of the moderate radiation dose level provided in the CRT schedule (41.4 Gy), and the more recent availability of advanced radiation therapy techniques, such as IMRT–SIB with IGRT, which could allow a more safe dose escalation in CRT for esophageal cancer both in terms of PTV coverage and healthy tissue sparing, when compared to 3D-CRT. Although no formal comparisons are available between sequential boost *vs* SIB in CRT for esophageal cancer, both approaches up to 60 Gy or more, appeared to improve locoregional control and survival when compared to the more standard dose of 50 Gy in the definitive CRT for inoperable disease (22).

As in a previous phase III trial comparing a dose of 64.8 Gy *vs* 50.4 Gy using the traditional 2D technique combined with cisplatin and 5-Fluorouracil (23), also the more recently reported results of a phase III dose escalation study with IMRT-SIB up to 61.6 Gy combined with carboplatin and paclitaxel, reported no significant improvement in local control and survival over 50.4 Gy with an increased toxicity and treatment-related deaths in the high dose arm (24). Other clinical trials are ongoing and results are awaited to further evaluate the efficacy of radiation dose intensification strategy in CRT in esophageal cancer

In our modified-CROSS program the intensified radiation dose was adapted on the basis of tumor location; we provided a dose of 50–52.5 Gy/25 fractions to EGJ and thoracic esophagus, while a higher dose of 54 Gy/25 fractions, with definitive intent, to the cervical esophagus. The CT component was the same as the original CROSS trial. Overall, the treatment was well tolerated with an incidence of Grade 3 toxicity (mainly esophagitis and leukopenia) in 19% of patients; no Grade 4 toxicity was reported. This acceptable incidence in severe toxicity allowed a high compliance to dose intensification with 93% of patients completing IMRT with SIB at the planned doses and treatment time. However, a number of patients required either interruption or dose modifications of concomitant CT due to Grade 3 or persistent Grade 2 toxicities, resulting in a less favorable compliance. As result, 54% of patients received four or five cycles and 87% at least three cycles of chemotherapy (**Table 2**). Compared to our results, the original CROSS regimen appeared better tolerated with lower Grade 3 toxicity (7%); in addition, the CROSS study adherence to whole treatment regimen of CT and radiotherapy was 91 and 92%, respectively (4). On the other hand, we used not only a higher, intensified dose of radiation, but also more extensive PTVs including the abdominal celiac node stations in EGJ and lower esophagus tumor locations, which were not usually included in the CROSS trial. In addition, our patient population was overall older (median age 69 *vs* 60 years) if compared to CROSS trial and no-patient selection was planned in our cohort. Nevertheless, we didn’t report any Grade 4 toxicity or treatment related death, as in CROSS trial, thus confirming the feasibility of our modified-CROSS regimen in an unselected patient population when a careful clinical monitoring and dose adequacy of concurrent carboplatin and paclitaxel are performed. To note, the induction with a median of three cycles (range 2–4) of taxane-based CT (25, 26) in 11 patients and the traditional cisplatin-based (27) in four patients, respectively, did not significantly affect the compliance to the subsequent CRT for this subset of patients (data not reported). This observation is of interest in the perspective of neoadjuvant combined modality programs including induction CT, as those currently ongoing for gastric cancer (28, 29), with the aim to reduce the high metastatic risk of esophageal and GEJ tumors. Radiation-induced acute lung toxicity and acute cardiac toxicity have not been reported in our experience with the intensified radiation dose given with IMRT and SIB. Nabavizadeh et al. (10), in their series of 24 patients treated with a modified-CROSS regimen with an IMRT dose of 50.4 Gy, reported three cases of postoperative ARDS, possibly related to the larger volume of lung irradiation with IMRT compared with 3D-CRT technique. The risk of acute lung injury must be an alert in esophageal irradiation, in particular when intensified doses are given with IMRT and SIB modalities; therefore a greater accuracy in the treatment planning, with individualized dose constraints (V20, Mean Dose, V5) is recommended to minimize this risk (30).

Our modified-CROSS regimen demonstrated effective; 48% of patients reported a cCR and 26% had a cPR for an overall response rate in 74% of patients. When correlated to histologic type, cCR rates were similar in SCC (50%) and AC (45%). These data are consistent with the available data on clinical response to CRT for esophageal and EGJ cancers, with cCR rates ranging from 28 to 86% (31). Moreover, our cCR rate of 48% is well comparable with the more recent investigations of radiation dose escalation programs in CRT for locally advanced, inoperable disease. Welsh et al. (32) reported a cCR rate of 71% in a phase I–II trial of dose escalation with IMRT–SIB up to doses of 58.8–63 Gy/28 fractions in 44 patients with inoperable disease, which is significantly higher compared to 52.5–54 Gy/25 fractions in our intensified IMRT–SIB program in more limited, resectable disease. Also in this study, AC and SCC histologic types had similar cCR rates, suggesting a clinical activity of intensified radiation doses in both histologic types. These favorable outcomes of dose escalation in CRT were confirmed by other Asian studies in advanced esophageal cancer, although most patients in these series had SCC histology (33, 34). However, the impact of clinical response, and in particular of the cCR, in disease control and survival needs to be investigated further. The available data on the association of cCR and pCR after CRT and surgery suggested a limited correlation, with approximately 31% of cCR corresponding to pCR after surgery (31). Nevertheless the evaluation of clinical response to CRT using computed tomography, EUS, endoscopy with biopsy remains an essential component in the clinical practice for subsequent treatment after CRT in esophageal and EGJ cancer (13, 20). The evaluation of clinical response is evolving with the support of PET–CT and the ongoing investigations on its role in the staging and restaging of disease before and after CRT should improve further the assessment of clinical response (35, 36).

On this basis, 34 (49%) responsive of 69 treated patients were selected for surgery at our MDT meetings after the modified-CROSS program. All 34 patients received the IMRT–SIB with a dose of 50 Gy (six patients) or 52.5 Gy (28 patients). Radical esophagectomy with negative margins (R0) was achieved in 30 of 32 (94%) operated patients (two patients were unresectable at surgery). This data is well comparable to that reported in the CRT arm of the original CROSS trial confirming the favorable impact of CRT on tumor and lymph-node response and resectability. Our modified CROSS regimen appeared effective in terms of pathologic response, with an overall pCR rate of 48%, compared to 29% of the original CROSS trial. A better pCR rate was also found in the subset of SCC (64 *vs* 49%) and AC patients (28 *vs* 23%). This data was also supported by the high rate of major pathologic response (TRG1 + TRG2) reported in 65% of our patients. In addition, most recurrences occurred at distant sites and only one patient had a local recurrence. Although these data need to be regarded with caution due to the small number of patients, they suggest the efficacy of our modified CROSS regimen.

Our incidence of postoperative complications (21%) after CRT is consistent with those reported in phase III trials (4, 7). Most patients (59%) had a minimally invasive esophagectomy at an experienced, high-volume surgical department for esophageal cancer and our 30-days postoperative mortality rate of 6% (two patients) was well comparable to that reported in the CROSS trial (6%). However, we reported one more patient who died of complications 58 days after surgery. These data underline the necessity of a careful patient selection for surgery after this intensive preoperative CRT program.

The analysis of survival was influenced by the limited follow-up (median 12 months, range 6–25) and the small series of patients in our study. However, the 2-year OS rate of 81% is of interest and is well comparable to 2-year OS of 67% of the CRT arm in the CROSS trial. Also, the 2-year PFS of 54% was similar to the DFS rate of CROSS indicating a promising benefit of our moderately intensified IMRT–SIB dose. Nevertheless, no difference in PFS was reported in the subset of the 34 operated patients (intention to treat analysis) when compared by histology; in spite of a higher pCR rate in SCC (71%) compared to AC (28%), we did not observe a higher benefit in survival for SCC as reported in CROSS trial. A similar benefit of CRT for both histologic types was also reported by Welsh et al. (32) in their IMRT dose escalation program; they suggested a possible greater benefit with higher radiation doses for patients with AC. This could be a significant data because of the prevalence of AC in Western Countries and the emerging interest in radiation dose escalation programs in CRT for esophageal and EGJ cancer.

Interestingly, there was no difference in the 2-year DFS for patients who received surgery compared to those non-operated after CRT. The major reasons for non-operation were cCR in patients with cervical esophagus (10%) and patient decision, shared with MDT, in case of cCR (14%) for the other tumor locations. While the non-operation option in patients with cervical tumors was expected because of the higher IMRT–SIB dose of 54 Gy with definitive intent, this option remains to be defined in cCR patients with other locations receiving 50–52.5 Gy with preoperative intent. The 2-year overall PFS in the subset of cCR patients, including non-operated, was significantly better when compared to non-complete responsive patients (75 *vs* 30%), and this data is in line with the emerging interest in non-operative approach for selected responsive patients (37–40). Our results in terms of PFS also show that most part of the disease progression occurred at distant sites. These data and the possible feasibility of induction CT could support further investigations on integrated programs which include a more effective systemic CT component in preoperative CRT. Further dose escalation over 54 Gy with definitive intent for cervical esophageal cancer remains questionable because of the controversial available results in oncological outcome, morbidity, and mortality even with the use modern and more advanced radiation techniques (22, 24, 32–34), and it should be further investigated.

Our study has some limitations. This is a retrospective study including a limited number of evaluated patients. The intensified dose levels that patients received as well as the addition of induction CT were made on an individual basis shared by the MDT. In addition to the limited number of patients, the short follow-up time limits the subset analysis for outcome in operated and non-operated patients beyond 2 years. Therefore a prospective study is needed to confirm these data.

In conclusion, this retrospective study reported favorable results in safety and feasibility of a preoperative CRT with IMRT–SIB dose intensification. The toxicity was acceptable allowing a high compliance to intensified radiation dose although dose reduction or delay in carboplatin/paclitaxel CT was needed in some patients. The high rate of cCR and pCR suggested that this moderately intensified treatment program is effective in the preoperative treatment and, in selected responsive patients, in the non-operative approach of esophageal and GEJ cancer. The 2-year survival rates were promising. While there is an emerging interest in the integration of preoperative CRT with a more effective systemic therapy component, our study is an early attempt at exploring the effects of a modified-CROSS regimen with IMRT and SIB dose intensification and its possible integration with induction CT. A prospective collaborative study is planned to confirm these observations.

## Data Availability Statement

The raw data supporting the conclusions of this article will be made available by the authors, without undue reservation.

## Ethics Statement

The studies involving human participants were reviewed and approved by the Internal Review Board CRO IRCCS Aviano. The patients/participants provided their written informed consent to participate in this study.

## Author Contributions

RI conceived and designed the study, and collected and sorted the data. RI and FN contributed to data collection. ADP and RI wrote the manuscript. RP and other surgeons had a huge role in patient treatment. JP performed the statistical analysis. All authors contributed to the article and approved the submitted version.

## Funding

This work was partially supported by the Italian Ministry of Health (Ricerca Corrente) [no grant number provided].

## Conflict of Interest

The authors declare that the research was conducted in the absence of any commercial or financial relationships that could be construed as a potential conflict of interest.
