# Supplementary material for: Feasibility and Oncological Outcome of Preoperative Chemoradiation With IMRT Dose Intensification for Locally Advanced Esophageal and Gastroesophageal Cancer
Source: Front Oncol. 2021 Feb 18;11:626275. doi: 10.3389/fonc.2021.626275 (PMC7930569; doi:10.3389/fonc.2021.626275)
Supplement: Supplementary file 1 [file DataSheet_1.docx]

**Supplementary documents**

Figure S1. Dose Volume Histogram (DVH) among treatment plan for EGJ adenocarcinoma (Sievert 1) reported in Figure 1.


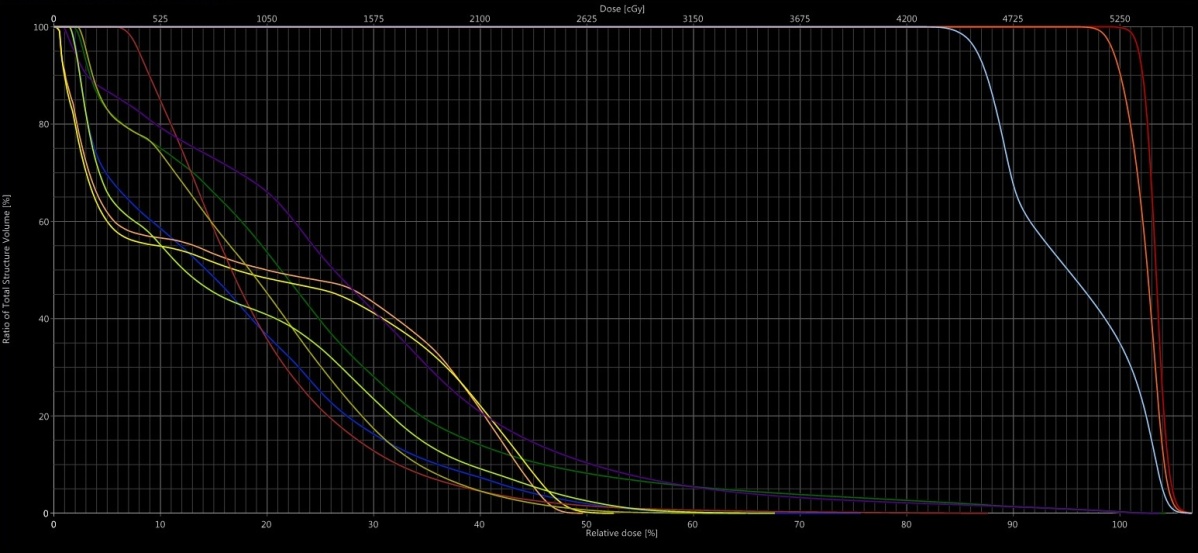


This example demonstrates the excellent coverage of PTV 52.5Gy (orange line) including GTV (red line): V95%≈98% and PTV 45Gy (light blue line): V95%≈98%, and the dose levels to OARs Right Lung (green line): V20≈15%, Mean dose≈13 Gy, V5≈75%; Left Lung (blue line): V20≈8%, Mean dose≈9 Gy, V5≈58%; Hearth (brown line) Mean dose≈10 Gy; Liver (purple line): V30≈5%, Mean dose≈14 Gy; Spinal cord (light orange line): Max dose 26 Gy;Spinal cord prv (yellow line): Max dose 27 Gy.

Table S1: Dose constraints used in treatment planning for IMRT dose intensification in preoperative CRT of esophageal and gastroesophageal cancer (11,30)

| - **Site** | - **Dose limits** |
| --- | --- |
| - Lungs | V20≤20%  V40≤10%  V5≤50%  Mean dose≤20 Gy |
| - Cord | Max Dose ≤40 Gy ( closer to 38 Gy preferred) |
| Heart | V30≤30% (closed to 20% preferred)  Mean≤25 Gy |
| Left Kidney and Right Kidney, separated | V20<30%  Mean dose≤18 Gy |
| Liver | V30≤20%  Mean dose≤25 Gy |
| Bowel | V45≤195cc |
